# Can we check serum lithium levels less often without compromising patient safety?

**DOI:** 10.1192/bjo.2021.1027

**Published:** 2021-12-17

**Authors:** Adrian H. Heald, David Holland, Michael Stedman, Mark Davies, Chris J. Duff, Ceri Parfitt, Lewis Green, Jonathan Scargill, David Taylor, Anthony A. Fryer

**Affiliations:** Department of Diabetes and Endocrinology, Salford Royal NHS Foundation Trust, Salford, UK, and The School of Medicine and Manchester Academic Health Sciences Centre, The University of Manchester, Manchester, UK; The Benchmarking Partnership, Alsager, Cheshire, UK; Res Consortium, Andover, UK; Res Consortium, Andover, UK; School of Medicine, Keele University, Keele, Staffordshire, UK, and Department of Clinical Biochemistry, University Hospitals of North Midlands NHS Trust, Stoke-on-Trent, Staffordshire, UK; Department of Clinical Biochemistry, University Hospitals of North Midlands NHS Trust, Stoke-on-Trent, Staffordshire, UK; St. Helens & Knowsley Teaching Hospitals NHS Trust, Whiston Hospital, Prescot, UK; Department of Clinical Biochemistry, The Royal Oldham Hospital, The Northern Care Alliance, Manchester, UK; Institute of Psychiatry, London, UK; School of Medicine, Keele University, Keele, Staffordshire, UK, and Department of Clinical Biochemistry, University Hospitals of North Midlands NHS Trust, Stoke-on-Trent, Staffordshire, UK

**Keywords:** Lithium, level, testing, interval, safety

## Abstract

**Background:**

Lithium is viewed as the first-line long-term treatment for prevention of relapse in people with bipolar disorder.

**Aims:**

This study examined factors associated with the likelihood of maintaining serum lithium levels within the recommended range and explored whether the monitoring interval could be extended in some cases.

**Method:**

We included 46 555 lithium rest requests in 3371 individuals over 7 years from three UK centres. Using lithium results in four categories (<0.4 mmol/L; 0.40–0.79 mmol/L; 0.80–0.99 mmol/L; ≥1.0 mmol/L), we determined the proportion of instances where lithium results remained stable or switched category on subsequent testing, considering the effects of age, duration of lithium therapy and testing history.

**Results:**

For tests within the recommended range (0.40–0.99 mmol/L categories), 84.5% of subsequent tests remained within this range. Overall, 3 monthly testing was associated with 90% of lithium results remaining within range, compared with 85% at 6 monthly intervals. In cases where the lithium level in the previous 12 months was on target (0.40–0.79 mmol/L; British National Formulary/National Institute for Health and Care Excellence criteria), 90% remained within the target range at 6 months. Neither age nor duration of lithium therapy had any significant effect on lithium level stability. Levels within the 0.80–0.99 mmol/L category were linked to a higher probability of moving to the ≥1.0 mmol/L category (10%) compared with those in the 0.4–0.79 mmol/L group (2%), irrespective of testing frequency.

**Conclusion:**

We propose that for those who achieve 12 months of lithium tests within the 0.40–0.79 mmol/L range, the interval between tests could increase to 6 months, irrespective of age. Where lithium levels are 0.80–0.99 mmol/L, the test interval should remain at 3 months. This could reduce lithium test numbers by 15% and costs by ~$0.4 m p.a.

## Background

Lithium was first found to have an acute antimanic effect in 1948,^1^ with further corroboration in the early 1950s.^2^ It took some time for lithium to become the standard treatment for relapse prevention in bipolar affective disorder, following the publication of early trials of lithium treatment in the 1960s.^3^ It was licensed for use in bipolar disorder by the US Food and Drug Administration in 1970.

The efficacy of lithium treatment in reducing relapses in bipolar disorder was confirmed in the largest randomised controlled trial of lithium for maintenance treatment of bipolar disorder to date.^4^ This showed that in patients stabilised on quetiapine during an acute phase of bipolar disorder (depression, mania or missed episode), switching to lithium significantly increased time to recurrence of any mood, manic or depressive event compared with switching to placebo. This study was notable in that it did not employ an enrichment design for lithium responsiveness in the acute phase.

Lithium is viewed as the first-line long-term treatment for prevention of relapse and hospital admission in people with bipolar disorder and is recommended by the UK National Institute for Health and Care Excellence (NICE)^5^ as well as in clinical practice guidelines in the USA, Canada, Japan, The Netherlands, Australia and New Zealand, and by the International Society for Bipolar Disorders.^6–8^ Lithium has other roles in psychiatry, including as an augmenting agent to antidepressants in unipolar depression.^9^

## Testing

Although lithium prescribing is declining, in October 2020, 72 000 prescriptions for lithium carbonate were issued in England.^10^ If one assumes that each prescription is for one patient, this suggests that around 70 000 patients are currently being prescribed lithium and thus require regular blood level monitoring in view of the narrow therapeutic window and risk of toxicity.

The recommended interval for monitoring of serum lithium levels varies according to the patient context. When lithium therapy is initiated, NICE guidelines recommend monitoring at weekly intervals until a stable lithium level is established.^5^ Subsequently, it is recommended that serum lithium levels be monitored on a 3 monthly basis for the first year of treatment, increasing to 6 monthly intervals for people under 65 years of age, with the intervals remaining at 3 months for people 65 years of age or older. More frequent monitoring may be required for a variety of reasons, including dose or formulation changes, changes in other medications or intercurrent illness. In particular, it is recommended that individuals with potentially toxic serum lithium concentrations (>1.4 mmol/L) should have serial daily lithium measurements taken to ensure rapid elimination of lithium.^11^

We have shown in other areas, including monitoring of HbA1c, thyroid function and cholesterol, that clinical laboratory test-requesting patterns are highly variable and that conformity to the guidance is suboptimal.^12–14^ We also showed that this is true for serum lithium monitoring,^15^ and that high variability in serum lithium testing between UK general practices is linked to an increased rate of hospital psychiatric admission.^16^ We are not aware of any prior research investigating the association between lithium testing interval and maintenance of serum levels within recommended ranges with a view to whether this interval could be extended in some cases.

In this study, our aims were to examine the factors associated with the likelihood of maintaining serum lithium levels within the recommended therapeutic range and to look at the stability of lithium levels between blood tests. We used request data for clinical laboratory serum lithium tests collected from three large UK centres with varying approaches to managing patients with bipolar disorder and ordering lithium testing.

## Method

### Data collection and categorisation

Data on all lithium requests received by the clinical biochemistry departments at the University Hospitals of North Midlands, Salford Royal Foundation Trust and Pennine Acute Hospitals NHS (National Health Service) Trust between 2012 and 2018 were extracted from the respective laboratory information and management systems (total 49 584 requests) as previously described.^15^ We collected data on test result, date of test, age, sex and source of request (general practice, hospital or community out-patient centre, accident and emergency department, or hospital in-patient unit).

As described previously,^15^ we excluded those requests related to people with a single lithium request, those initiating lithium therapy in the final year of data collection and those under the age of 18, leaving a data-set of 46 555 requests from 3371 individuals.

Serum lithium concentrations were grouped into four categories: (a) LO, <0.40 mmol/L (below the British National Formulary (BNF) recommended therapeutic range); (b) BNF/NICE, 0.40–0.79 mmol/L (within BNF and/or NICE ranges); (c) BNF/NICE relapse, 0.80–0.99 mmol/L (within BNF range, but where more frequent monitoring is recommended when a previous relapse has occurred or where there are subsyndromal symptoms); and (d) HI, ≥1.00 mmol/L (above both BNF and NICE ranges, where there is an increased risk of toxicity).^5,11,17^

Results in the recommended therapeutic range (0.40–0.99 mmol/L) were defined as ‘within accepted range’. For those patients whose current results were within accepted range, we examined the associations of (a) patient age, (b) duration of lithium treatment and (c) result history within the previous 12 months with the proportion of tests that remained within accepted range at subsequent testing. We then examined the association of ‘duration to the next lithium test’ with the proportion of results remaining within the accepted range.

This study is part of a quality improvement programme to increase the quality of laboratory test requesting. Hence, it includes a service evaluation and audit of local practice against the guidelines outlined by NICE and the BNF,^5,17^ with a view to increasing implementing quality improvements to enhance the clinical laboratory service. Accordingly, this study was not considered to be research using the decision tool provided by the UK Health Research Authority (http://www.hra-decisiontools.org.uk/research/)^18^ and did not require NHS Research Ethics Committee review. All data extracted from laboratory information and management systems were anonymised.

### Data analysis

All data manipulation was carried using Power Pivot in Microsoft Excel.

#### Interval between tests

The total numbers of tests carried out in each monthly period were calculated. These were then split into two groups based on whether the current result was inside or outside the accepted range (0.40–0.99 mmol/L). If the current test was within the accepted range, the analysis considered the percentage of the next tests’ results that remained within the accepted range.

#### Age

According to current guidelines,^5^ age is considered important with relation to testing frequency. Therefore, patient age at the time of the test was allocated into 10 year bands, and the proportion of patients with test results in accepted range was evaluated for each band.

#### Duration of lithium therapy

Those patients who had results in the first year of the data-set were taken as already being on lithium and so were excluded from this analysis. For all the others, it was assumed that lithium had been initiated during the study period. This was deemed to be a reasonable assumption, as the number of existing patients with an interval between tests of greater than 12 months was shown to be less than 2%. The first recorded test (after excluding those whose first test was in the first year) was used as the date of entry into the study. The time intervals since entry into the study were grouped into years, and the percentage within accepted range of subsequent tests was evaluated for each group.

#### Previous test result history

For each patient and test result, we identified the tests carried out within the previous 13 months (12 months + 1 month to allow for some appointment flexibility). The proportion of these prior results falling within the accepted range was taken as the measure of patient test result history.

### Impact of interval between tests for those outside and within the therapeutic range

For each of the above indicators, we examined how the proportion within the accepted therapeutic range was linked to the interval between tests according to: (a) age, consolidated into people aged <65 years and ≥65 years old at time of test; (b) duration of therapy, stratified into those in their first year after initiation and those on treatment for over 1 year; and (c) previous test result history, aggregated into those with no test results outside the therapeutic range within the prior 13 months (100% within range) and those with <100% of prior results within range.

### Alternative test frequency modelling

Based on the above findings, we made recommendations for test requesting frequency that would potentially reduce the total amount of testing, while improving the probability of patients remaining within the therapeutic range. We evaluated the effect of these recommendations on the total number of tests required.

### Ethics

As we anonymised patient data, it was not deemed necessary to seek ethics approval for this study. However, approval was sought and obtained from the respective research and innovation departments at each of the three hospitals participating in the study.

## Results

### Percentage of lithium results falling within defined serum concentration bands and cumulative values

**Fig. 1:**
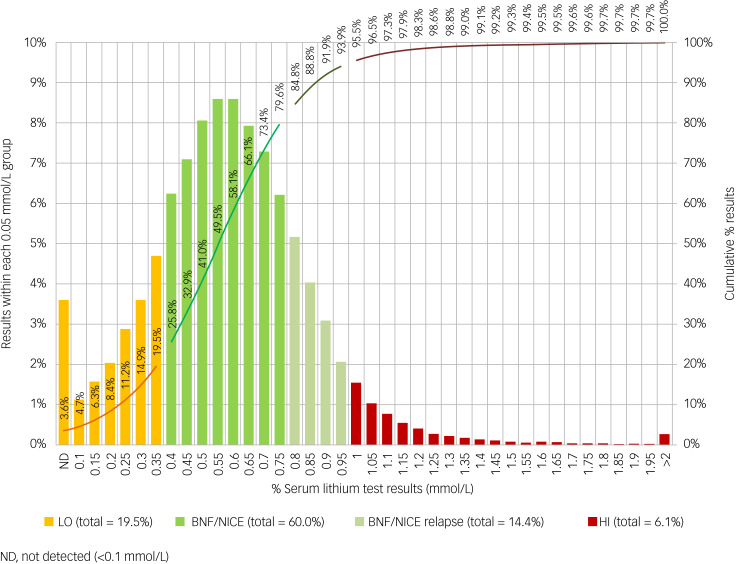
Percentage of total lithium results falling within given bands and cumulative values. LO, <0.40 mmol/L; BNF/NICE, 0.40–0.79 mmol/L; BNF/NICE relapse, 0.80–0.99 mmol/L; HI, ≥1.00 mmol/L.

Figure 1 shows the distribution of all lithium test results. In total, 19.5% of results were below the accepted range (LO), 74.4% were within the accepted range (60.0% BNF/NICE and 14.4% BNF/NICE relapse) and 6.1% were high at 1.0 mmol/L or above (HI).

### Flow of lithium level from a test at one point in time to the next test

**Fig. 2:**
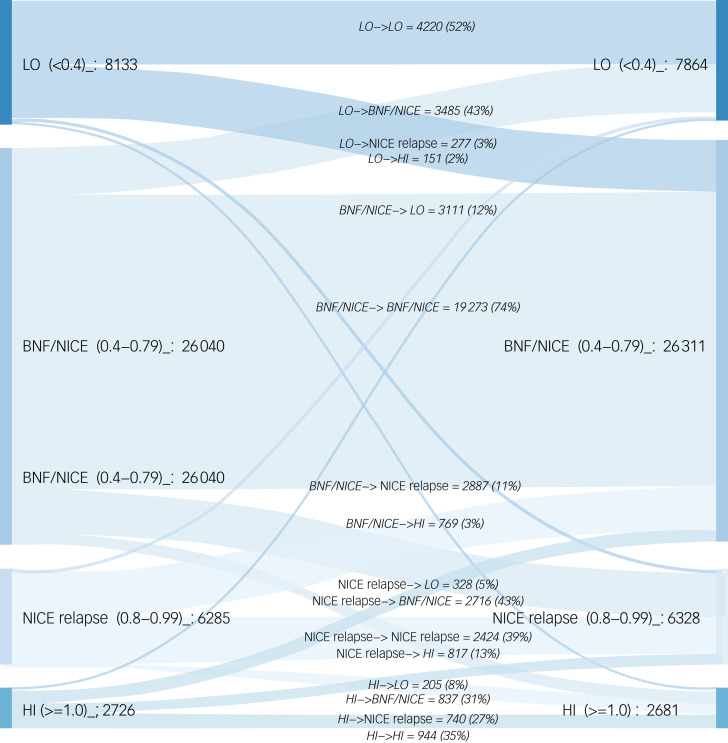
Sankey diagram illustrating the flows between initial and subsequent lithium test results by category. LO, <0.40 mmol/L; BNF/NICE, 0.40–0.79 mmol/L; BNF/NICE relapse, 0.80–0.99 mmol/L; HI, ≥1.00 mmol/L.

We then examined the proportion of cases in each of the four lithium level categories based on initial and subsequent lithium concentrations. In particular, we examined the proportion of cases where a result changed category between the initial and subsequent test. This was illustrated using a Sankey diagram (Fig. 2). For both those aged <65 years and those aged ≥65 years, the flows between serum lithium groups were similar irrespective of age group (data not shown).

For the 0.40–0.79 mmol/L category, in 74% of instances, the subsequent lithium result was in the same category as the initial result, suggesting that a stable level had been achieved. In those with low initial levels (<0.40 mmol/L), 43% moved to the 0.40–0.79 mmol/L category. Among cases with high levels (≥1.0 mmol/L), 35% remained in the same category, 27% moved to the 0.80–0.99 mmol/L category and 31% moved to the 0.40–0.79 mmol/L category.

The results shown in Fig. 2 thus demonstrate that whereas the majority of patients remain within the same class of lithium level, a significant proportion of people do shift category from one test point to the next.

### Percentage of overall test results by interval between tests

**Fig. 3:**
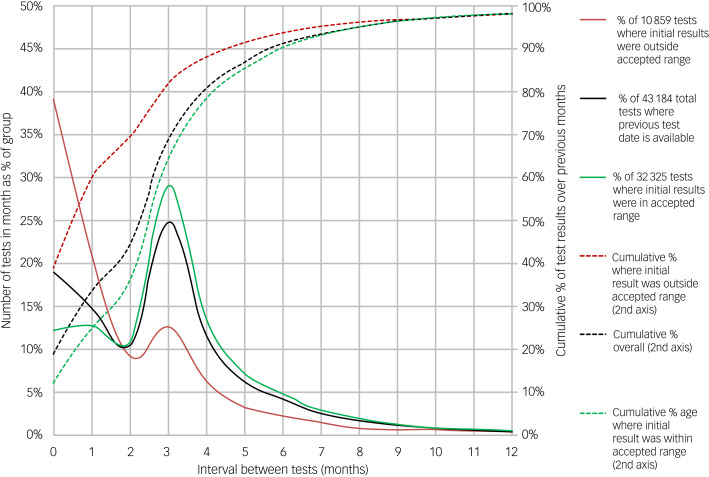
Relative frequency plot by number of months interval to previous test for all results and also split by results falling within and outside accepted range. Cumulative percentages are shown on second (right-hand) axis.

Figure 3 shows a relative frequency plot of the proportion of total serum lithium tests by the number of months to the next test. It also shows the cumulative percentage of tests performed with increasing interval between tests. The time interval to the subsequent test was used, as this should be determined by the result of the initial test. To highlight the effects, the data were also stratified by whether the initial result was within or outside the accepted range (0.40–0.99 mmol/L). In instances where the initial lithium test result was within the accepted range, there was a significant peak at 3 months but no peak at 6 months. Moreover, 65% of tests were carried out within 3 months, but also ~55% of these tests within 3 months were carried out within 2 months or less. In cases where the initial result was outside the expected range, there was a higher proportion of re-requests within 2 months, although there was still a noticeable peak at 3 months and 18% remained not retested even by this stage.

### Effects of age, duration of lithium therapy and previous test result history on the percentage of results remaining within the accepted range

**Fig. 4:**
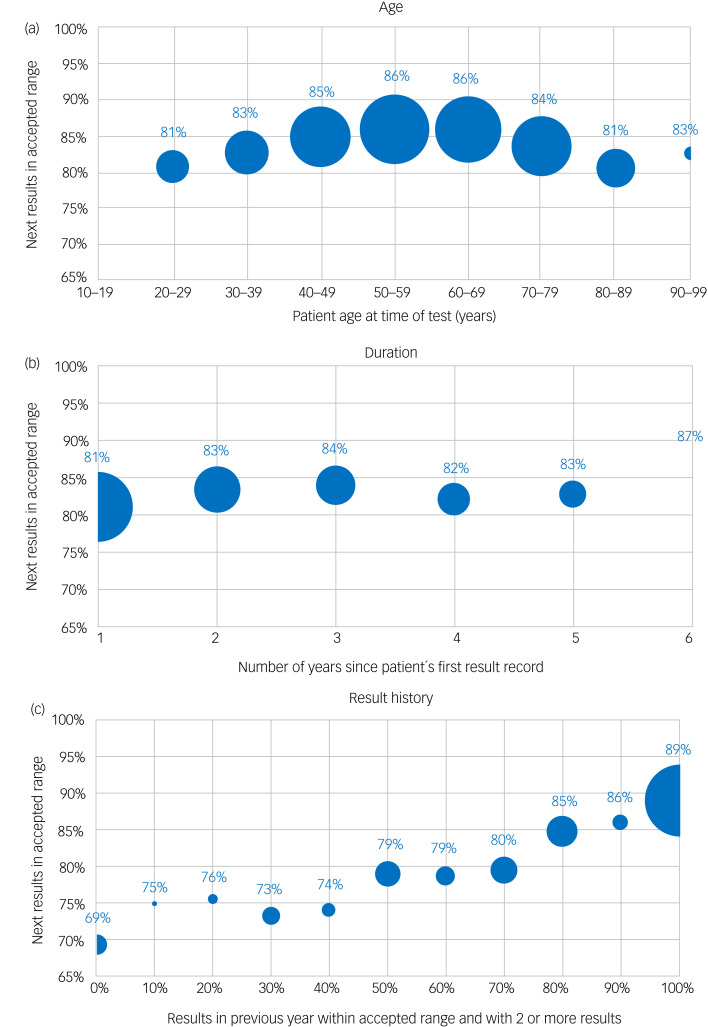
Impact of chosen factors on percentage of current total tests falling within accepted range (0.40–0.99) where previous test was within the accepted range shown by: (a) age group; (b) duration in years since starting tests; and (c) previous test history (percentage of test results within the accepted range).

The proportion of cases where the initial result was ‘within range’ which had a subsequent test result ‘within range’ was highest among patients aged 50–59 years (86%; Fig. 4a). This proportion was lower in both lower and higher age groups, with the lowest percentage being in those aged 20–29 years (81%). However, there was generally relatively little clinically relevant impact of age on the probability of remaining ‘within range’.

There was no discernible link between the duration of lithium therapy and the proportion of results remaining within the accepted range (Fig. 4b). However, the proportion of test results within the accepted range in the previous 12 months appeared to be directly associated with the likelihood of subsequent results remaining within the accepted range (Fig. 4c). For those with no previous results outside the recommended range in the prior 12 months, 89% of subsequent results remained on target, compared with fewer than 75% for those with <50% of prior results within the recommended range.

These results highlight that age and duration of lithium therapy have relatively little impact on the proportion of results remaining within range, whereas prior test result history in relation to stability of lithium level in the previous 12 months has a marked effect.

### Association between testing interval and proportion remaining within accepted range

**Fig. 5:**
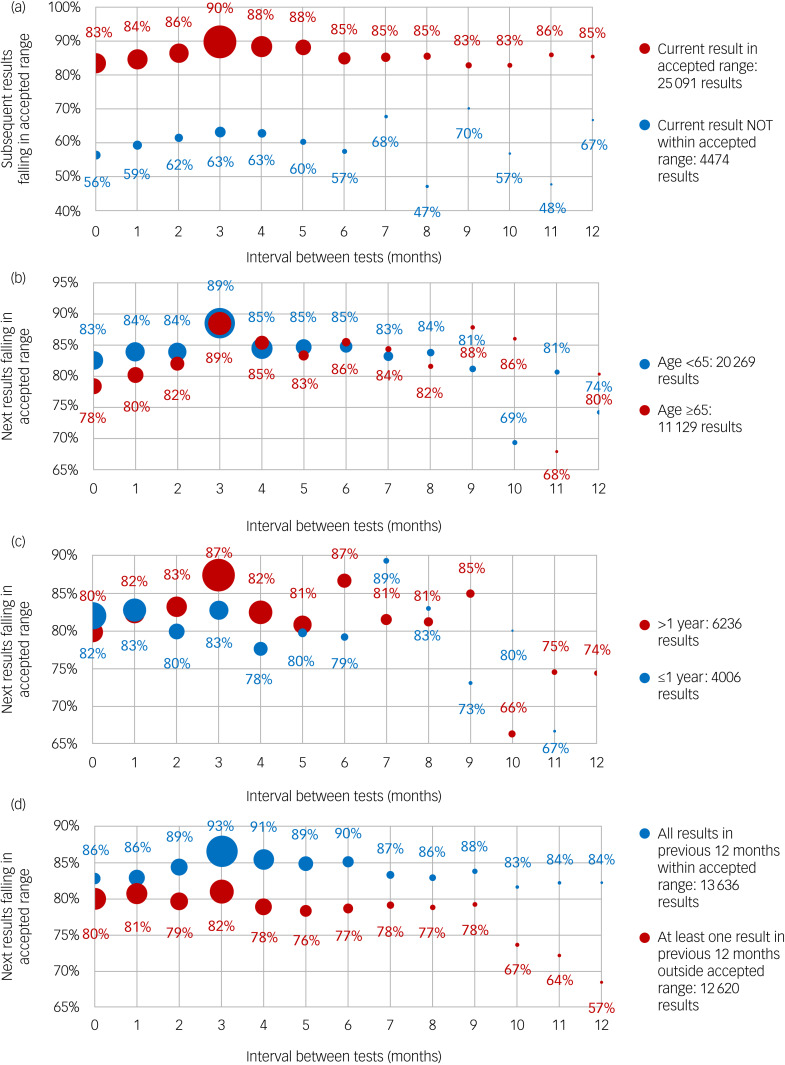
Duration in months between test by various factors on percentage of current total tests falling within accepted range by period since previous test was within accepted group. (a) Overall, considering those whose current test results were within and outside the accepted range. (b) Age group <65 or older. (c) Duration of therapy >1 year or less. (d) Previous test history: 100% results in previous 12 months were within acceptable range or <100% of results within range.

Figure 5 examines the association between the proportion of cases with an initial result within the recommended range (0.40–0.99 mmol/L) whose subsequent test results remained within range and the interval between the initial and subsequent test. In this analysis, we again explored the influence of age, duration of lithium therapy and previous test result history.

Overall, this showed that the proportion of tests remaining within range remained at 85–90% for test intervals of 3–6 months, with a peak at 3 months. However, this dropped below 85% for intervals of less than 2 months or more than 9 months (Fig. 5a). Among cases where the initial result was outside the accepted range, the proportion of subsequent tests within range was lower at 60%, irrespective of interval between tests, although the number of cases within this group was smaller.

#### Age

The proportion remaining within the recommended range was highest for those tested at 3 months, both for those aged <65 years and for those ages ≥65 years (Fig. 5b). Overall, there was little difference among age groups in the association between the proportion remaining within range and the testing interval.

#### Duration of therapy

Comparison of those in their first year with those on lithium therapy for >1 year showed little effect of duration of therapy on the association between the testing interval and the proportion remaining within range (Fig. 5c).

#### Previous test result history

For most test intervals, the lithium test result history in the previous 12 months was clearly associated with the proportion of subsequent test results within range, with patients with 100% of results on target doing much better than those with <100% results on target (Fig. 5d). Among those with all previous results within range, the proportion remaining within range if tested at 3–6 months was similar (93–90%); however, this dropped below 90% for those tested both more or less frequently.

### Probability of next lithium test result being ≥1.0 mmol/L

**Fig. 6:**
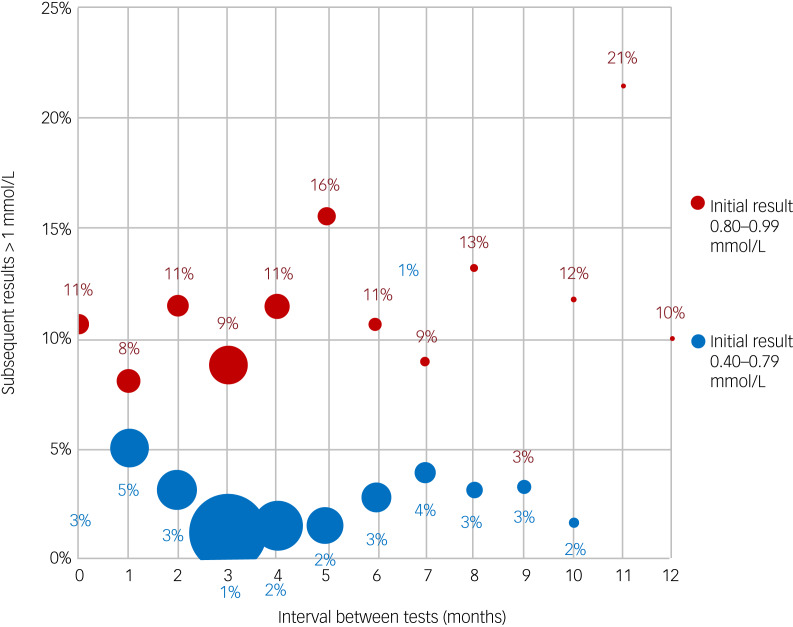
Risk of next lithium result being ≥1.0 mmol/L for period to the next test, splitting the current results between 0.40–0.79 (BNF/NICE) and 0.80–0.99 (BNF/NICE relapse).

Figure 6 shows the link between the testing interval and the proportion of patients with a subsequent test result of 1.0 mmol/L or more, stratified by their current result (0.40–0.79 mmol/L *v*. 0.80–0.99 mmol/L). As expected, those with a current result of 0.80–0.99 mmol/L had a higher probability of the subsequent result being ≥1.0 mmol/L (~10% compared with <5% if the initial result was 0.40–0.79 mmol/L) regardless of testing interval. We suggest that this group may warrant more frequent testing.

### Suggested changes to testing frequency

Based on the above findings, we suggest alternative recommendations for test frequency that enhance the likelihood of remaining on target without compromising patient safety. We also examine the impact of such changes on test numbers.

The results of initial (current) tests were allocated to three groups as follows.

- Outside therapeutic range (<0.40 mmol/L or ≥1.0 mmol/L): the recommendation is that patients with these results are retested as soon as any dose adjustment can titrate through (within 4 weeks). Patients with extremely high results (>1.4 mmol/L) should be retested immediately, as per the guidelines on suspected lithium toxicity.^5^
- Within therapeutic range with at least one result within the previous year outside therapeutic range: these patients should remain on the current 3 month retest interval.
- Within therapeutic range with all results in the previous year also within therapeutic range: the test interval for these patient can be extended to 6 months with no increase in the risk of subsequent tests being outside therapeutic range.

To evaluate how these new rules might affect the total number of tests needed during a given period, we allocated current tests to the above classes and summed the overall test period for each of the patients (between their first and their last test). We assumed that the proportions between classes would remain the same as the testing interval changed, and then applied the new intervals to the total number of tests in each class to calculate a new overall proposed time for patients to be tested. The ratio between the new estimated period and current time period gives an estimate of the potential change in the number of tests in a given period.

Table 1 shows how adjusting the period to the next test would affect the total number of tests being carried out within the same period as that assessed during this study. Existing practice demonstrates 46 555 lithium tests over a total period of 133 870 patient months, measured as the sum of the time gap between all initial and subsequent tests. These were split across the three classes outlined above, with 26% falling outside the range, 36% within range but with at least one previous result outside, and 38% with no previous results falling outside the range. Applying the test frequency recommendation for each class, we found that the overall patient time covered was 126% of the current overall time. This means that to cover the same test period as covered by the current regime, 21% fewer tests would be required if the recommended alternative testing regime was adopted.

**Table 1:** Effect of implementing the proposed recommended testing frequency

	Initial test not in accepted range	Initial test in accepted range and at least one result outside accepted range in previous year	Initial test in accepted range and all results within accepted range in previous year	Overall
Current number of tests	11 921	16 899	17 735	46 555
Current total period between initial and subsequent tests (months)	31 035	37 763	65 072	133 870
Current average interval between tests (months)	2.60	2.23	3.67	2.88
Proposed test interval (months)	1.0	3.0	6.0	
Total duration with proposed interval (months)	11 921	50 697	106 410	169 028
% change in total duration *v*. current practice	38.4%	134.3%	163.5%	126%
Estimated number of tests using recommended intervals	9441	13 384	14 046	36 872

In October 2020, 72 000 prescriptions for lithium carbonate were issued. Based on this, it is estimated that around 72 000 patients are on lithium carbonate and being tested every 2.88 months (as derived from the average interval in Table 1), suggesting that approximately 300 000 lithium tests are carried out nationally each year. The general population base covered by this study is 2% of the national population, so the 46 555 tests included here over 7 years also would also correspond to around 321 000 tests/year nationally. A 21% reduction would save approximately 67 500 tests per annum. At an estimated cost of more than £8/test for phlebotomy, sample transport and laboratory testing, this would save the NHS circa £0.54 m/year.

A further option is that only patients in the current BNF/NICE recommended range of 0.40–0.79 mmol/L should move to 6 month intervals, and that those within the BNF/NICE relapse range of 0.80–0.99 mmol/L would remain on 3 monthly testing. In this case, 3573 tests would move from 6 to the 3 month interval, and the savings in the same period would fall to ~15% of the current test volume, equating to a potential saving of approximately £0.4 million p.a.

## Discussion

In this quality improvement initiative, we examined the effects of various factors on the probability of serum lithium results remaining within the recommended therapeutic range. Based on these findings, we suggest a possible alternative testing regime for those on lithium therapy. However, we are not attempting to make any substantive recommendations, rather to inform opinion leaders as to what we have found. The intention is that our findings relevant to lithium testing schedules will be taken into account in the synthesis of evidence for any future recommendations.

### Percentage of results within the accepted range

A total of 74.4% of lithium results were within the accepted range in relation to agreed limits that are applied in the UK (Fig. 1).^5,17^ This suggests that the current testing protocols are largely effective but that there is potential to increase the interval between tests in certain circumstances. Overall, a 3 month test interval was associated with 90% of lithium test results staying on target, compared with a 6 month interval where 85% test results stayed on target (Fig. 5). This can be seen in the context of the study of Tondo et al (2017),^19^ where it was found that the average lithium level was extraordinarily stable over many years, because lithium dosage was reduced with age.

### Flow of lithium level from a test at one point in time to the next

As shown in Fig. 2, we found that although the majority of patients remained within the same lithium level category (irrespective of age), significant numbers of patients did move categories on subsequent testing. It makes sense that testing frequency should focus on those that move from one lithium level category to another. Importantly, 27 300/32 325 (84.5%) of lithium test results previously within the BNF/NICE or BNF/NICE relapse^5,17^ categories remained within one or the other of these categories on subsequent testing. This may suggest that there is an opportunity to revise the recommended testing frequency, which is already relatively onerous in a complex patient group, without compromising patient safety.

We agree that there is a small drop in the proportion remaining in range with longer intervals between tests. In our experience in this and other contexts,^12–14,16,20,21^ we find that a longer interval is associated with poorer outcomes. We are not aiming to address the underlying causes for this here; it is indeed a complex area beyond the scope of this work but will include factors associated with patients, healthcare professionals and systems. We looked at age, duration of treatment and prior result history (Fig. 5) in the present work, though stratifying patients with intervals beyond 6 months would result in groups that are too small to draw any definite conclusions.

### What influences the next lithium result?

We showed that 70% of lithium tests are carried out by 3 months after the last test (Fig. 3). This is supported by our previous work.^15^ We also found that for a given retest interval, age and duration have little impact on the proportion remaining within range (Fig. 4). However, prior history in relation to stability of lithium level in the previous 12 months has a marked effect and could potentially be used as a discriminator. To our knowledge, this is the first time this effect has been described. This may reflect those individuals who are engaged with healthcare services and compliant with treatment, for whom the likelihood of continued engagement is high.

It is interesting that age was not a noticeable factor. This calls into question using age as a differentiator for determining frequency of testing as described in the NICE guidance.^5^

The association between a 3 month test interval and the highest proportion of tests within range (Fig. 5) is likely to be related, at least partly, to organisational and patient concordance factors^22^ rather than any specific calendar effect. In other words, a fixed regime for blood tests is more beneficial than a more *ad hoc* test schedule. Thus, some general practices and mental health services may be better organised to deliver check-ups at the required interval. We and others have noted this regular pattern of testing for other conditions such as diabetes.^12,16,20,21,23^

As might be expected, the results shown in Fig. 6 indicate that the 0.80–0.99 mmol/L group had a higher risk of moving to a level of ≥1.00 mmol/L at subsequent testing, regardless of test interval, and so it is reasonable to propose that this group should be tested at 3 monthly intervals, as per the existing guidance.^5,17^

Many tests were repeated outside the expected frequencies, indicating the need for additional work to minimise inappropriate testing. This is a common finding in this and other conditions.^12–15^ Hence, many unnecessary lithium tests may be being conducted in people with levels already on target.^7,24^

We found that ~19% of tests outside the target range had not been followed up within 3 months. This may be related to a combination of lithium treatment being discontinued in these individuals or to the person not turning up for any further lithium checks. Freeing up valuable resources by stopping ‘unnecessary’ testing of people with stable within-range lithium levels to focus on people whose last level was out of range would be a sensible strategy. The support of clinical laboratory professionals in freeing up general practitioner time by supporting the management of test monitoring may be one way to facilitate this.

### What should be the target range for lithium?

Previous studies have looked at what should be the target range for lithium. Nolen and Weisler in 2013^25^ in a *post hoc* analysis reported that times to recurrence of any manic or depressive event were significantly longer in the lithium 0.6–1.2 mmol/L group versus placebo and versus lithium <0.6 mmol/L, with no differences between lithium <0.6 mmol/L and placebo. They recommended that lithium should be dosed high enough to achieve plasma levels ≥0.6 mmol/L in order to achieve an effect in the prevention of both manic and depressive recurrences of bipolar disorder. As shown in Fig. 2, we found that the great majority of target-range lithium test results were followed by another test result in the accepted range.

### Patient safety

Risk factors for the development of lithium-induced renal impairment include the length of duration of therapy and increasing age, as well as episodes of over-dosage or elevated lithium levels. The available evidence indicates therefore that stability of lithium levels in an agreed therapeutic range is a significant factor in ensuring patient safety.

### How can we reduce unnecessary lithium testing?

We propose that for individuals who have achieved 12 months of stable lithium tests within the range 0.40–0.79 mmol/L, it would be reasonable to increase the interval between tests to 6 months, irrespective of age, while regular monitoring of renal function is maintained (as per guidance).^5,17^ Where lithium levels are in the 0.80–0.99 mmol/L range, the retest interval should be maintained at 3 months. Ideally, individuals should have had more than one lithium level within the therapeutic range in the previous 12 months before the index measurement.

As shown in Table 1, adoption of our new testing frequency proposals could potentially avoid up to 21% of the estimated 321 000 of current annual lithium tests, these being reduced by 67 500. At an estimate of £8 per lithium test, including phlebotomy/nursing time and transport, that could save £0.54 million/year. However, there is also a much greater but less easily quantifiable benefit in focusing retesting on the 26% of test results falling outside the target range such that these are completed within 1 month rather than the current 2.6 month average, thereby more than halving the 25% of time patients might be outside the target area.

Reducing the consequences of high and low serum lithium levels through increasing the frequency of testing in those patients with the 26% of tests falling outside the range is likely to have substantial benefits (financial and patient).

### Strengths and limitations

Our study used data on large numbers of patients across three UK sites and highlighted the potential of laboratory-based data to examine longitudinal monitoring in a range of conditions.^12–15^ However, unlike some other approaches, our method did not enable us to determine specific information from clinical laboratory records on the reason for each lithium test request or the underlying primary psychiatric diagnosis. We accept that the people who turn up for 3 monthly tests are more concordant with the treatment programme in general and are more likely to turn up for blood tests and to take the medication as prescribed, and that this is a potential confounding factor. We do not have any clinical details as to the relationship between phases of illness and an individual's lithium levels.^26^

In an important review of conformity to lithium monitoring across 38 UK mental health trusts by Collins et al,^27^ the authors showed that around 60% of patients on lithium therapy had a primary diagnosis of bipolar disorder, 25% had unipolar depression and 11% had psychotic spectrum disorder. Although we recognise that this is a potentially important limitation, the recommendations for lithium monitoring within national and international guidance are consistent regardless of indication.^5^ Furthermore, many local Shared Care Agreements focus on lithium therapy rather than specific primary diagnosis.^28,29^ Finally our analysis did not look at serum creatinine/eGFR, calcium (bone profile) or thyroid hormone status. These will be subjects of further analyses.

### Clinical implications

We propose that for individuals who have achieved 12 months of stable lithium tests within the range 0.40–0.79 mmol/L, it would be reasonable to increase the interval between tests to 6 months, irrespective of age (as is currently recommended by NICE^5^), while simultaneous monitoring of renal function is maintained. Where lithium levels are 0.80–0.99 mmol/L, the test interval should be maintained at 3 months. With this regime, patient safety can be maintained while optimising resource utilisation.

## Data Availability

Any requests for data extracts will be considered by A.H.H. as the corresponding author. No patient-identifiable data were used in the analysis.
